# Validation of a semiconductor next-generation sequencing assay for the clinical genetic screening of *CFTR*

**DOI:** 10.1002/mgg3.149

**Published:** 2015-04-16

**Authors:** Daniel Trujillano, Maximilian E R Weiss, Julia Köster, Efstathios B Papachristos, Martin Werber, Krishna Kumar Kandaswamy, Anett Marais, Sabrina Eichler, Jenny Creed, Erol Baysal, Iqbal Yousuf Jaber, Dina Ahmed Mehaney, Chantal Farra, Arndt Rolfs

**Affiliations:** 1 Centogene AGRostock, Germany; 2 Pathology and Genetics Department, Dubai Genetic and Thalassemia Center, Dubai Health AuthorityDubai, United Arab Emirates; 3 Clinical and Chemical Pathology Department, Faculty of Medicine, Cairo UniversityCairo, Egypt; 4 Department of Pathology & Laboratory Medicine, American University of Beirut Medical CenterBeirut, Lebanon; 5 Albrecht-Kossel-Institute for Neuroregeneration, Medical University RostockRostock, Germany

**Keywords:** *
CFTR
*, cystic fibrosis, Ion Torrent, molecular diagnostics, next-generation sequencing, validation

## Abstract

Genetic testing for cystic fibrosis and *CFTR*-related disorders mostly relies on laborious molecular tools that use Sanger sequencing to scan for mutations in the *CFTR* gene. We have explored a more efficient genetic screening strategy based on next-generation sequencing (NGS) of the *CFTR* gene. We validated this approach in a cohort of 177 patients with previously known *CFTR* mutations and polymorphisms. Genomic DNA was amplified using the Ion AmpliSeq™ *CFTR* panel. The DNA libraries were pooled, barcoded, and sequenced using an Ion Torrent PGM sequencer. The combination of different robust bioinformatics tools allowed us to detect previously known pathogenic mutations and polymorphisms in the 177 samples, without detecting spurious pathogenic calls. In summary, the assay achieves a sensitivity of 94.45% (95% CI: 92% to 96.9%), with a specificity of detecting nonvariant sites from the *CFTR* reference sequence of 100% (95% CI: 100% to 100%), a positive predictive value of 100% (95% CI: 100% to 100%), and a negative predictive value of 99.99% (95% CI: 99.99% to 100%). In addition, we describe the observed allelic frequencies of 94 unique definitely and likely pathogenic, uncertain, and neutral *CFTR* variants, some of them not previously annotated in the public databases. Strikingly, a seven exon spanning deletion as well as several more technically challenging variants such as pathogenic poly-thymidine-guanine and poly-thymidine (poly-TG-T) tracts were also detected. Targeted NGS is ready to substitute classical molecular methods to perform genetic testing on the *CFTR* gene.

## Introduction

Pathologic genetic alterations in the cystic fibrosis transmembrane conductance regulator (*CFTR/ABCC7*; MIM #602421), cause the impairment of chloride transport in epithelial cells that line the passageways of the lungs, pancreas, sweat glands, and vas deferens in men (O’Sullivan and Freedman 2009). Mutations in *CFTR* are associated with cystic fibrosis (CF; MIM #219700), which is the most common life-threatening autosomal recessive genetic disorder (Farrell 2008), but also male infertility due to congenital bilateral absence of the vas deferens (CBAVD; MIM #277180), idiopathic chronic pancreatitis (MIM #167800), and bronchiectasis (MIM #211400), among others *CFTR-*related disorders (Dequeker et al. 2009; Whitcomb 2012).

The challenge in *CFTR* genetic screening resides on its high allelic heterogeneity, with more than 1900 sequence variants reported (Cystic Fibrosis Mutation Database, January 2015, http://www.genet.sickkids.on.ca) since its discovery 25 years ago (Kerem et al. 1989; Riordan et al. 1989; Rommens et al. 1989). Although one mutation (deltaF508) accounts for about 70% of CF alleles worldwide (Bobadilla et al. 2002), diverse heritages are reflected for the *CFTR* gene and distributed with varying frequencies among populations often complicating genetic analysis (Estivill et al. 1997). The current guidelines of the American College of Medical Genetics and Genomics (ACMG) recommend a panel of only 23 variants for population-based CF carrier screening (Watson et al. 2004), leaving the vast majority of possible genotype changes untested.

To date, the identification of *CFTR* pathologic variants relies on commercial tests that screen for specific common mutations and/or laborious direct DNA Sanger sequencing of the moderately large *CFTR* gene (27 exons) (Nakano et al. 2014). Yet, more effective next-generation sequencing (NGS) technologies are rapidly tested and introduced into clinical practice (Grosu et al. 2014). Here, we validated an NGS analysis pipeline based on the Ion Torrent PGM benchtop next-generation sequencer and the Ion AmpliSeq™ *CFTR* Panel (Life Technologies, Carlsbad, CA) combined with robust bioinformatics tools for *CFTR* genetic screening. In order to investigate its applicability to clinical genetic diagnostics we performed a broad analysis of *CFTR* variants in 177 previously characterized patients with diverse CF and *CFTR*-related phenotypes. We were able to identify genetic alterations in *CFTR*, including single nucleotide variants (SNV), insertions and deletions (InDels) and structural variants (SV). Strikingly, a seven exon spanning deletion as well as several more technical challenging variants such as pathogenic poly-thymidine-guanine and poly-thymidine (poly-TG-T) tracts were detected.

## Materials and Methods

### Patients

In total 177 CF and *CFTR-*related patients were recruited into the study during the time period between April and September 2014. All had previously undergone conventional genetic diagnosis by Sanger sequencing of all *CFTR* exons and, if negative, Multiplex Ligation-dependent Probe Amplification (MLPA) was also applied. Based on the recommendation of their physicians, all participants requested genetic testing for the *CFTR* gene. Our cohort included patients from Australia (1), Austria (1), Brazil (3), Canada (6), Denmark (1), Dubai (12), Egypt (45), Finland (3), Germany (2), India (4), Iran (9), Iraq (1), Italy (1), Jordan (2), Lebanon (24), Malta (1), Mexico (1), Oman (1), Pakistan (5), Panama (3), Qatar (1), Saudi Arabia (1), Sweden (15), United Arab Emirates (19), United States of America (13), Yemen (2), being 57 of them females, 69 males (51 of unknown gender), with an average age of 32 years old. All samples were anonymized and blindly sequenced and analyzed. The study was approved by the Ethical Commission of the Faculty of Medicine of the University of Rostock and informed consent was signed by all contributing subjects prior to the *CFTR* genetic testing.

### DNA extraction

DNA was isolated from Ethylenediaminetetraacetic acid (EDTA) blood using two automated procedures. The spin−column-based extraction was performed on QIAcube instrument with QIAamp DNA Blood Mini QIAcube Kit (Qiagen, Valencia, CA) following the manufacturer instructions. Alternatively the QIAsymphony DSP DNA Mini Kit (Qiagen) on the QIAsymphony instrument was used to purify the DNA from blood. Following extraction all DNA samples were stored at −20°C. Prior to the analysis the DNA quality and concentration was determined photometrically (OD_260_/OD_280_ 1.8–2.0).

### Amplicon library construction

The target regions in the *CFTR* gene were amplified using the Ion AmpliSeq™ *CFTR* Panel (Life Technologies). It consists of two primer pools (102 amplicons) that target the entire coding region, including 10–20 bp of intronic flanking sequences around all coding exons, of the gene. In order to amplify each library 4 *μ*L of 5X Ion AmpliSeq™ HiFi mix, 10 *μ*L of 2X Ion AmpliSeq™ primer pool (two of them in separate wells for each sample), 10 ng of gDNA per reaction (2 *μ*L of 5 ng/*μ*L stock), and 4 *μ*L of nuclease free water were mixed together. Following temperature profile was applied to the final 20 *μ*L of PCR mixture: 99°C for 2 min; 99°C for 15 sec, 60°C for 4 min (19 cycles); with a final hold at 10°C. Then primer sequences were partially digested, and adapters and barcodes ligated to the amplicons as described in Ion AmpliSeq™ library preparation manual. Each library was marked with a unique adapter provided in Ion Xpress™ barcode adapters 1–96 Kit (Life Technologies). Purified libraries were quantified with the Qubit® 2.0 fluorometer (Life Technologies) using the Qubit® dsDNA HS assay kit, diluted to ∼100 pmol/L and combined in equimolar proportion. Freshly prepared library stock dilutions were used on the same day for the preparation of enriched, template-positive ion sphere particles (ISPs). Automated protocols were run on the Ion OneTouch™ 2 System and the Ion OneTouch™ ES Instrument (Life Technologies) according to the version of the user guide and using the 200 bp chemistry kits.

### Sequencing on the Ion Torrent platform

All barcoded samples were sequenced on the PGM (Life Technologies) with 318 chips taking up to 48 samples on a single chip per sequencing run. Chip loading procedure was performed twice according to the user guide for the on Ion PGM™ sequencing 200 kit v2.

### Data analysis

Raw sequence data analysis, including base calling, demultiplexing, alignment to the hg19 human reference genome (Genome Reference Consortium GRCh37), and variant calling, were performed using the Torrent Suite Software v.4.0.2 (Life Technologies). For the variantCaller plugin we used the optimized parameters for the *CFTR* panel. Variants were annotated using Annovar (Wang et al. 2010) and in-house ad hoc bioinformatics tools. Alignments were visually verified with the Integrative Genomics Viewer v.2.1 (Robinson et al. 2011) and Alamut v.2.2 (Interactive Biosoftware, Rouen, France).

Variant analysis was performed without bias with a cascade of filtering steps previously described (Walsh et al. 2010). The reference sequence used for *CFTR* was NM_000492.3. All candidate variants were required on both sequenced DNA strands and to account for ≥20% of total reads at that site with a minimum depth of coverage of 80X. Common polymorphisms (≥5% in the general population) were discarded by comparison with dbSNP138, the 1000G (January 2015, http://www.1000genomes.org), the Exome Variant Server (January 2015, http://evs.gs.washington.edu), and an in-house exome variant database to filter out both common benign variants and recurrent artifact variant calls. However, as these databases also contain known disease-associated mutations, all detected variants were compared to our internal mutation database (CentoMD®) and HGMD® to directly identify and annotate changes previously described in the literature as definitely and likely pathogenic, uncertain, and neutral variants.

The 95% confidence intervals (CI) were calculated by statistical inference using the standard deviation (SD) (Mattocks et al. 2010; Chan et al. 2012). In instances where there were no false positives (SD = 0), the 95% CI were produced with the Wilson score method (Newcombe 1998).

### Evaluation of the pathogenicity of the variants

Evaluation of the pathogenicity of the variants not previously described in the literature and absent in the CentoMD® and HGMD® databases was performed with the following criteria. Mutations predicted to result in a premature truncated protein: nonsense, frameshift mutations, and large genomic rearrangements, as well as canonical splice site mutations were classified as definitely pathogenic. Missense variants were considered a priori unclassified sequence variants (UCV) and their potential pathogenicity was evaluated taking into consideration the biophysical and biochemical difference between wild type and mutant amino acid, the evolutionary conservation of the amino acid residue in orthologs (Tavtigian et al. 2006), a number of in silico predictors (Sift, Polyphen, Mutation taster and Condel), and population data. Then UCV were classified into three groups: likely pathogenic, neutral and variants of uncertain significance when previously conflicting information has been published about their functionality. Noncanonical splicing variants were analyzed using Alamut version 2.2 (Interactive Biosoftware), a software package that uses different splice site prediction programs to compare the normal and variant sequences for differences in potential regulatory signals.

## Results

### Sequencing statistics

The Ion AmpliSeq™ *CFTR* Panel (Life Technologies) generates 102 amplicons of 150 bp on average, that cover all targeted coding exons and exon–intron boundaries (including 10–20 bases of flanking sequences around all targeted coding exons) of the *CFTR* gene. It has been designed to yield sequence coverage redundancy with overlapping amplicons across exons. Sequencing of the *CFTR* gene in the 177 patients generated a mean of 90,650 reads per patient. On average, 98% of these reads mapped to the targeted regions of *CFTR*. An evenly distributed mean depth of coverage of 852X for *CFTR* was achieved (Table1). Ninety-four percent of the targeted base pairs of *CFTR* were covered by more than 100 reads. To determine if coverage was substantially lower for any region, we calculated the proportion of base pairs that were captured by <50 reads, which is the minimum that we required to perform variant calling. The proportion of these poorly covered regions accounted for 2.35% of *CFTR* targeted base pairs, being all of them randomly spread over intronic regions at the ends the amplicons and sequencing reads.

**Table 1:** Average sequencing quality control and coverage statistics of *CFTR* across the 177 patients.

177 patients	QC-passed reads	Mapped reads	On target	Mean depth (X)	Uniformity
Average	90,651	90,336	0.98	852	0.95
SD	65,669	65,304	0.01	609	0.05

	% ALL target bases covered				
	= 0X	≥20X	≥50X	≥100X	≥200X
Average	0.70	98.83	97.65	94.63	86.97
SD	1.15	1.97	3.90	8.39	16.93

**Figure 1:**
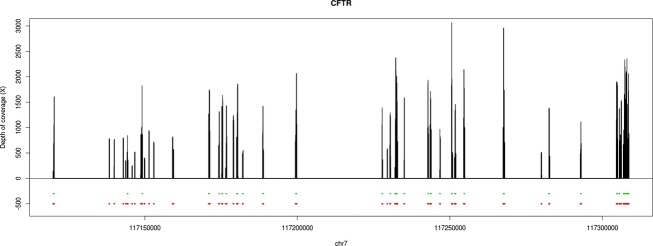
Representation of the average depth of coverage of *CFTR* across the 177 patients. Green lines represent the exons of the gene. Red lines represent the amplicons of the assay.

From these data, we can conclude that all samples were uniformly covered at depths that in all cases exceed by far the minimum coverage required for reliable variant calling (Fig.1). The minor differences between samples were neutralized by the excessive overall coverage achieved by the assay. The sequence quality metrics of this data warrant a confident detection of variants in all patients.

### Detection of *CFTR* variants

The selection of the samples for this study was carried out with the idea to include as many different types of *CFTR* variants as possible, to simulate a real-world diagnostics scenario, so that we could test the performance of the NGS assay for different types of genetic variation. To assess the sensitivity of the assay we blindly inspected all mapped sequence reads from the 177 samples with previously defined sequence variants analyzed with the conventional diagnostic workflow.

We identified 630 of 667 previously known mutations and variants in their correct zygosity status, including SNVs, InDels, and large SVs achieving a sensitivity of 94.45% (95% CI: 92% to 96.9%). The 37 false negatives accounted for a total of four unique variants: c.744-37_744-34delATTA (seen in 2 patients), c.744-9_744-6del (seen in 30 patients), c.1647T>G (seen in 1 patient), and c.3718-2531A>G (seen in 4 patients). All of them are intronic benign variants, except c.1647T>G which has been previously reported as pathogenic. All false negative variants were consequence of the absence sequencing reads in their loci in the affected patients. However, these regions were identified by the bioinformatics pipeline and automatically reported as target regions with low or inexistent sequence coverage that required Sanger sequencing repeats for gap filling the NGS data.

To assess the specificity of the assay across the targeted bases of the *CFTR* gene, we evaluated all sequenced positions previously screened by Sanger sequencing. Genotype data were available across the 177 patients for a total of 1,823,100 sites within the targeted regions of *CFTR*. Specificity of detecting nonvariant sites from the *CFTR* reference sequence was 100% (1,822,433/1,822,433; 95% CI: 100% to 100%).

The positive predictive value of the assay, calculated as [number of true positives] / [number of true positives + number of false positives], was 100% ([630] / [630 + 0]; 95% CI: 100% to 100%). The negative predictive value of the assay, calculated as [number of true negatives] / [number of true negatives + number of false negatives], was 99.99% ([1,822,432] / [1,822,433 + 37]; 95% CI: 99.99% to 100%).

**Figure 2:**
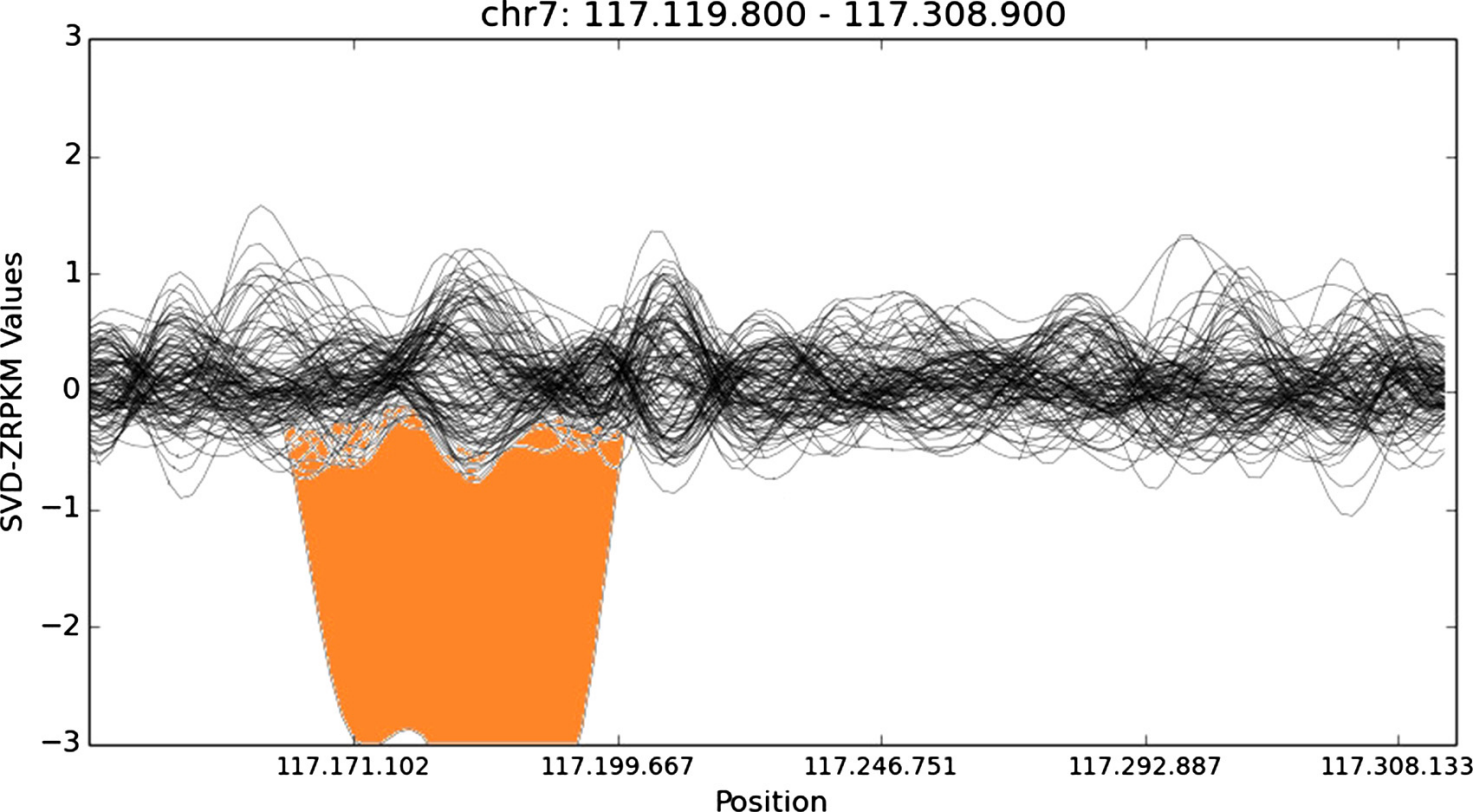
Detection of a large deletion by normalized depth of coverage analysis. Representation of the SVD-ZRPKM values calculated by Conifer.

We also inspected the *CFTR* sequencing depth profile of all the patients with the aim to detect large SVs. Noteworthy, a previously described homozygous large deletion spanning exons 4–11 was detected in one of the patients (Fig.2), confirming the previous MLPA results (i.e. 0 false positive calls).

Finally, we also inspected our samples for the poly-TG-T (c.1210–34TG[11–13]T[5–9]) haplotype using a custom script. By doing this, we were able to determine the exact TG-T haplotype of each sample, including different twelve TG10-T5, eleven TG11-T5, and seven TG12-T5 pathogenic haplotypes associated with CBAVD.

An overview of the definitely pathogenic mutations, likely pathogenic mutations, uncertain, and neutral variants in these samples, as confirmed by conventional Sanger sequencing and MLPA, is listed in Table2.

**Table 2:** CFTR variants identified in the 177 patients.

Location	cDNA change	Protein change	dbSNP138	HGMD	Variant type	Hom/Het of Total
Intron 02	c.164+12T>C	p.(?)	rs121908790	CS982112	Definitely pathogenic	1/0 of 177
Exon 03	c.178G>T	p.(E60*)	rs77284892	CM920141	Definitely pathogenic	0/1 of 177
Exon 03	c.202A>G	p.(K68E)	–	CM972935	Definitely pathogenic	0/1 of 177
Intron 03	c.274-6T>C	p.(?)	rs371315549	CS930762	Definitely pathogenic	0/1 of 177
Exon 04	c.416A>T	p.(H139L)	rs76371115	CM001649	Definitely pathogenic	1/0 of 177
Exon 04	c.454A>G	p.(M152V)	–	CM931142	Definitely pathogenic	1/0 of 177
Exon 06	c.650A>G	p.(E217G)	rs121909046	CM972939	Definitely pathogenic	0/1 of 177
Exon 08	c.902A>G	p.(Y301C)	rs150691494	CM990360	Definitely pathogenic	0/1 of 177
Exon 08	c.1040G>C	p.(R347P)	rs77932196	CM900044	Definitely pathogenic	1/0 of 177
Exon 08	c.1043T>A	p.(M348K)	rs142920240	CM930106	Definitely pathogenic	0/1 of 177
Exon 11	c.1397C>G	p.(S466*)	rs121908805	CM940251	Definitely pathogenic	2/0 of 177
Exon 11	c.1399C>T	p.(L467F)	rs1800089	CM063898	Definitely pathogenic	0/3 of 177
Exon 11	c.1521_1523del	p.(F508del)	rs199826652	CD890142	Definitely pathogenic	13/10 of 177
Exon 12	c.1624G>T	p.(G542*)	rs113993959	CM900049	Definitely pathogenic	1/1 of 177
Exon 12	c.1646G>A	p.(S549N)	rs121908755	CM900050	Definitely pathogenic	0/1 of 177
Exon 12	c.1647T>G	p.(S549R)	rs121909005	CM900052	Definitely pathogenic	5/2 of 177
Exon 13	c.1684G>A	p.(V562I)	rs1800097	CM990362	Definitely pathogenic	1/0 of 177
Intron 13	c.1766+3A>G	p.(?)	–	CS971651	Definitely pathogenic	0/1 of 177
Exon 14	c.1911del	p.(Q637fs)	–	CD920846	Definitely pathogenic	1/0 of 177
Exon 14	c.2051_2052delinsG	p.(K684Sfs*38)	–	CX931110	Definitely pathogenic	1/1 of 177
Exon 14	c.2353C>T	p.(R785*)	rs374946172	CM941979	Definitely pathogenic	1/0 of 177
Intron 15	c.2620-15C>G	p.(?)	rs139379077	CS004690	Definitely pathogenic	0/4 of 177
Intron 16	c.2657+5G>A	p.(?)	rs80224560	CS900235	Definitely pathogenic	1/1 of 177
Exon 17	c.2758G>A	p.(V920M)	rs373885282	CM980351	Definitely pathogenic	0/1 of 177
Exon 17	c.2834C>T	p.(S945L)	–	CM930123	Definitely pathogenic	1/1 of 177
Intron 18	c.2988+1G>A	p.(?)	rs75096551	CS971653	Definitely pathogenic	1/0 of 177
Exon 20	c.3154T>G	p.(F1052V)	–	CM930125	Definitely pathogenic	0/1 of 177
Exon 20	c.3209G>A	p.(R1070Q)	rs78769542	CM930128	Definitely pathogenic	2/1 of 177
Exon 21	c.3409A>G	p.(M1137V)	–	CM931152	Definitely pathogenic	0/1 of 177
Exon 22	c.3705T>G	p.(S1235R)	rs34911792	CM930133	Definitely pathogenic	0/1 of 177
Intron 22	c.3718-24G>A	p.(?)	rs374013084	CS086376	Definitely pathogenic	0/1 of 177
Exon 23	c.3846G>A	p.(W1282*)	rs77010898	CM900061	Definitely pathogenic	0/2 of 177
Exon 23	c.3872A>G	p.(Q1291R)	–	CM940279	Definitely pathogenic	1/0 of 177
Exon 24	c.3909C>G	p.(N1303K)	rs80034486	CM910076	Definitely pathogenic	1/1 of 177
Intron 26	c.4242+13A>G	p.(?)	rs76179227	CS056085	Definitely pathogenic	0/1 of 177
Exon 27	c.4333G>A	p.(D1445N)	rs148783445	CM962488	Definitely pathogenic	0/1 of 177
3′UTR	c.*1043A>C	p.(?)	rs10234329	CR133159	Definitely pathogenic	0/1 of 177
Exon 11	c.1542_1543del	p.(Y515*)	–	–	Likely pathogenic	1/0 of 177
Exon 27	c.4250del	p.(E1417fs)	–	–	Likely pathogenic	0/1 of 177
5′UTR	c.-45T>A	p.(?)	–	–	Uncertain	0/1 of 177
5′UTR	c.-4G>C	p.(?)	rs369326781	–	Uncertain	0/2 of 177
Intron 01	c.54-13C>G	p.(?)	–	CS920988	Uncertain	0/1 of 177
Intron 02	c.164+28A>G	p.(?)	rs34010645	CS040534	Uncertain	0/1 of 177
Exon 03	c.224G>A	p.(R75Q)	rs1800076	CM980331	Uncertain	0/2 of 177
Exon 03	c.227_228insT	p.(C76fs)	–	–	Uncertain	1/0 of 177
Exon 04	c.350G>A	p.(R117H)	rs78655421	CM900043	Uncertain	0/3 of 177
Exon 04	c.443T>C	p.(I148T)	rs35516286	CM920145	Uncertain	0/2 of 177
Exon 04	c.473G>C	p.(S158T)	–	CM055123	Uncertain	0/2 of 177
Exon 05	c.532G>T	p.(G178*)	–	–	Uncertain	1/0 of 177
Exon 07	c.844_845insA	p.(E282fs)	–	–	Uncertain	0/1 of 177
Exon 09	c.1132C>T	p.(Q378*)	–	–	Uncertain	0/1 of 177
Exon 11	c.1584G>A	p.(E528E)	rs1800095	CS014912	Uncertain	0/3 of 177
Exon 12	c.1670C>T	p.(S557F)	–	–	Uncertain	0/1 of 177
Exon 19	c.2991G>C	p.(L997F)	rs1800111	CM920171	Uncertain	0/2 of 177
Exon 19	c.3063_3068del	p.(I1023_V1024del)	–	–	Uncertain	0/1 of 177
Intron 19	c.3139+18C>T	p.(?)	rs147945812	–	Uncertain	0/1 of 177
Exon 22	c.3538del	p.(K1180fs)	–	–	Uncertain	0/1 of 177
Exon 22	c.3637del	p.(K1213fs)	–	–	Uncertain	0/1 of 177
Intron 26	c.4243-36del	p.(?)	–	–	Uncertain	0/1 of 177
5′UTR	c.-8G>C	p.(?)	rs1800501	–	Neutral	0/9 of 177
Intron 02	c.165-67A>C	p.(?)	rs376666464	–	Neutral	0/1 of 177
Exon 04	c.360G>A	p.(A120A)	rs1800077	–	Neutral	0/1 of 177
Intron 04	c.489+91A>G	p.(?)	rs56094102	–	Neutral	0/5 of 177
Intron 06	c.743+40A>G	p.(?)	rs1800502	–	Neutral	2/13 of 177
Intron 06	c.744-33_744-30delGATT	p.(?)	rs34543279	–	Neutral	17/13 of 177
Intron 07	c.869+11C>T	p.(?)	rs1800503	–	Neutral	23/22 of 177
Exon 10	c.1251C>A	p.(N417K)	rs4727853	–	Neutral	0/1 of 177
Exon 10	c.1265C>T	p.(S422F)	rs201880593	–	Neutral	0/1 of 177
Exon 10	c.1312A>G	p.(T438A)	rs201434579	–	Neutral	0/1 of 177
Intron 10	c.1393-61A>G	p.(?)	rs34855237	–	Neutral	33/45 of 177
Exon 11	c.1408G>A	p.(V470M)	rs213950	–	Neutral	61/64 of 177
Intron 12	c.1680-871A>G	p.(?)	–	–	Neutral	1/5 of 177
Intron 12	c.1680-870T>A	p.(?)	rs213965	–	Neutral	62/64 of 177
Exon 15	c.2562T>G	p.(T854T)	rs1042077	CS042144	Neutral	27/49 of 177
Exon 17	c.2793C>T	p.(F931F)	–	–	Neutral	0/1 of 177
Exon 17	c.2898G>A	p.(T966T)	rs1800109	–	Neutral	0/1 of 177
Intron 17	c.2909-71G>C	p.(?)	rs34830471	–	Neutral	0/7 of 177
Intron 19	c.3139+42A>T	p.(?)	rs28517401	–	Neutral	0/1 of 177
Intron 19	c.3139+89T>C	p.(?)	rs151033496	–	Neutral	0/1 of 177
Intron 19	c.3140-92T>C	p.(?)	rs4148717	–	Neutral	0/13 of 177
Intron 20	c.3367+37G>A	p.(?)	rs137854873	–	Neutral	0/1 of 177
Intron 20	c.3368-140A>C	p.(?)	rs213981	–	Neutral	13/44 of 177
Intron 20	c.3368-89G>C	p.(?)	rs139520130	–	Neutral	0/2 of 177
Intron 21	c.3469-65C>A	p.(?)	rs213989	–	Neutral	13/44 of 177
Exon 23	c.3870A>G	p.(P1290P)	rs1800130	–	Neutral	0/9 of 177
Intron 23	c.3873+117T>G	p.(?)	rs10155917	–	Neutral	0/2 of 177
Exon 24	c.3897A>G	p.(T1299T)	rs1800131	–	Neutral	0/1 of 177
Intron 24	c.3963+69A>G	p.(?)	–	–	Neutral	1/0 of 177
Intron 26	c.4243-53C>T	p.(?)	rs185664216	–	Neutral	1/1 of 177
Exon 27	c.4272C>T	p.(Y1424Y)	rs1800135	–	Neutral	1/1 of 177
Exon 27	c.4389G>A	p.(Q1463Q)	rs1800136	CS042143	Neutral	15/48 of 177
3′UTR	c.*1105C>T	p.(?)	–	–	Neutral	0/1 of 177
3′UTR	c.*1106A>C	p.(?)	–	–	Neutral	0/2 of 177
3′UTR	c.*1251C>T	p.(?)	rs1042180	–	Neutral	15/49 of 177

* NM_000492.3 for *CFTR*.

## Discussion

The accurate diagnosis of CF combines clinical evaluation, in particular medical symptoms of the CF phenotype and sweat test measurements, with *CFTR* genetic testing. To date, the molecular characterization of *CFTR* mutations in a given sample relies on commercial tests that screen for specific common mutations. Test panels range from 4 to 70 *CFTR* mutations and comprise technologies such as reverse dot blot INNO-LIPA CFTR (Innogenetics, Gent, Belgium), Cystic Fibrosis Genotyping Assay/OLA (Abbott, Chicago, IL), Elucigene CF-EU2 (Elucigene, Manchester, United Kingdom) and xTAG Cystic Fibrosis 71 kit v2 (Luminex, Austin, TX) among others. More recently an FDA approved NGS-based platform screening for 139 *CFTR* mutations from Illumina has been released (Grosu et al. 2014). The detection rate of these panels varies depending on the mutations included and the molecular heterogeneity of each population. For many patients with common *CFTR* mutations that are present in these commercial panels, there is no need for additional studies. However, the high heterogeneity of *CFTR* mutations in some CF populations and in *CFTR*-RD often requires the complete molecular screening of the 27 exons and the regulatory regions of *CFTR*, a putative costly and labor-intensive task.

The purpose of this study was to evaluate and establish a NGS workflow based on the Ion Torrent PGM benchtop next-generation sequencer (Life Technologies), as a routine method for comprehensive genetic screening of *CFTR* for CF and *CFTR*-related diagnostics. We show that it can be easily incorporated into clinical practice with low-cost and short turnaround time. In addition, this strategy offers a complete definition of the two genes, including the 23-mutation panel recommended by the ACMG, without the need, anymore, for stepwise testing and choosing which exon to sequence first.

While excluding carry-over estimation, specimen stability testing and intra or interassay precision assessment, we performed a comprehensive NGS versus Sanger genotype comparison that has statistically validated, in terms of both sensitivity and specificity, the Ion Torrent PGM benchtop NGS method for routine CF screening. The bioinformatics pipeline applied here proves high sensitivity, specificity and predictive values in detecting different classes of sequence variants. We are able to identify the most important genetic alterations in *CFTR*, including SNVs, InDels, and large SVs. Strikingly, a seven exon spanning deletion as well as several more technically challenging variants such as the pathogenic poly-TG-T haplotypes were detected.

Recently, different NGS platforms and genomic enrichment strategies have been tested for the identification of sequence variants in *CFTR,* demonstrating comparable performance in terms of both specificity, sensitivity, and time- and cost effectiveness with the assay described here (Trujillano et al. 2013; Grosu et al. 2014). In our case, we decided to adopt in our *CFTR* diagnostics workflow the PGM in combination with the Ion AmpliSeq™ *CFTR* Panel (Life Technologies), because it delivers fast TAT coupled with throughput flexibility, enabling rapid time-to-results in processing either a small or large number of samples. In addition, it offers fast library construction for affordable targeted sequencing of the *CFTR* gene, based on ultrahigh-multiplex PCR, requiring as low as 10 ng of input DNA. All these arguments make of this system a convenient NGS configuration easily adaptable by diagnostic labs, as an accurate, economical, and easy-to-implement end-to-end solution.

In routine diagnostics current stepwise Sanger sequencing and choosing which genetic region to sequence first, often becomes time consuming and expensive. Additionally, Sanger has been shown to be incomplete in terms of the identification of disease-causing variants and intricate *CFTR* regions such as poly-TG-T in comparison to NGS (Chen and Prada 2014). Our NGS-based strategy not only enables rapid time-to- highly accurate results in processing either small or large sample numbers, but also offers fast library construction for affordable targeted sequencing of the *CFTR* gene based on ultrahigh-multiplex PCR.

In summary, we are opening new diagnostic avenues to concurrently investigate different types of pathogenic sequence variants by presenting a NGS-based *CFTR* genetic screening workflow as a precise and economical alternative to conventional *CFTR* genetic testing in medical laboratories. This straightforward – one assay – approach offers high clinical convenience for the handling of CF genetic diagnostics, allowing test reporting 7 days after receiving the DNA samples.
